# Evaluation of feasibility phase of adaptive version of locally made bubble continuous positive airway pressure oxygen therapy for the treatment of COVID-19 positive and negative adults with severe pneumonia and hypoxaemia

**DOI:** 10.7189/jogh.13.06046

**Published:** 2023-11-24

**Authors:** Mohammod Jobayer Chisti, Ahmed Ehsanur Rahman, Taufiq Hasan, Tahmeed Ahmed, Shams El Arifeen, John David Clemens, Abu Sayem Mirza Md. Hasibur Rahman, Md. Fakhar Uddin, Md. Robed Amin, Md. Titu Miah, Md. Khairul Islam, Mohiuddin Sharif, Abu Sadat Mohammad Sayeem Bin Shahid, Anisuddin Ahmed, Goutom Banik, Meemnur Rashid, Md. Kawsar Ahmed, Lubaba Shahrin, Farzana Afroze, Monira Sarmin, Sharika Nuzhat, Supriya Sarkar, Jahurul Islam, Muhammad Shariful Islam, John Norrie, Harry Campbell, Harish Nair, Steve Cunningham

**Affiliations:** 1 International Centre for Diarrhoeal Disease Research, Bangladesh (icddr,b), Dhaka, Bangladesh; 2 Bangladesh University of Engineering and Technology (BUET), Dhaka, Bangladesh; 3 Center for Bioengineering Innovation and Design, Johns Hopkins University, Baltimore, Maryland, USA; 4 International Vaccine Institute, Seoul, Korea; 5 Line director, NCD, DGHS, Dhaka, Bangladesh; 6 DG, DGME, Dhaka, Bangladesh; 7 Dhaka Medical College Hospital (DMCH), Dhaka, Bangladesh; 8 Directorate General of Health Services, Ministry of Health and Family Welfare, Dhaka, Bangladesh; 9 NIHR Global Health Research Unit on Respiratory Health (RESPIRE), Usher Institute, The University of Edinburgh, Edinburgh, Scotland, UK

## Abstract

**Background:**

Bubble continuous positive airway pressure (bCPAP) oxygen therapy has been shown to be safe and effective in treating children with severe pneumonia and hypoxaemia in Bangladesh. Due to lack of adequate non-invasive ventilatory support during coronavirus disease 2019 (COVID-19) crisis, we aimed to evaluate whether bCPAP was safe and feasible when adapted for use in adults with similar indications.

**Methods:**

Adults (18-64 years) with severe pneumonia and moderate hypoxaemia (80 to <90% oxygen saturation (SpO_2_) in room air) were provided bCPAP via nasal cannula at a flow rate of 10 litres per minute (l/min) oxygen at 10 centimetres (cm) H_2_O pressure, in two tertiary hospitals in Dhaka, Bangladesh. Qualitative interviews and focus group discussions, using a descriptive phenomenological approach, were performed with patients and staff (n = 39) prior to and after the introduction (n = 12 and n = 27 respectively) to understand the operational challenges to the introduction of bCPAP.

**Results:**

We enrolled 30 adults (median age 52, interquartile range (IQR) 40-60 years) with severe pneumonia and hypoxaemia and/or acute respiratory distress syndrome (ARDS) irrespective of coronavirus disease 2019 (COVID-19) test results to receive bCPAP. At baseline mean SpO_2_ on room air was 87% (±2) which increased to 98% (±2), after initiation of bCPAP. The mean duration of bCPAP oxygen therapy was 14.4 ± 24.8 hours. There were no adverse events of note, and no treatment failure or deaths. Operational challenges to the clinical introduction of bCPAP were lack of functioning pulse oximeters, difficult nasal interface fixation among those wearing nose pin, occasional auto bubbling or lack of bubbling in water-filled plastic bottle, lack of holder for water-filled plastic bottle, rapid turnover of trained clinicians at the hospitals, and limited routine care of patients by hospital clinicians particularly after official hours.

**Discussion:**

If the tertiary hospitals in Bangladesh are supplied with well-functioning good quality pulse oximeters and enhanced training of the doctors and nurses on proper use of adapted version of bCPAP, in treating adults with severe pneumonia and hypoxaemia with or without ARDS, the bCPAP was found to be safe, well tolerated and not associated with treatment failure across all study participants. These observations increase the confidence level of the investigators to consider a future efficacy trial of adaptive bCPAP oxygen therapy compared to WHO standard low flow oxygen therapy in such patients.

**Conclusion:**

s Although bCPAP oxygen therapy was found to be safe and feasible in this pilot study, several challenges were identified that need to be taken into account when planning a definitive clinical trial.

Severe acute respiratory syndrome coronavirus 2 (SARS-CoV-2) caused a global pandemic primarily affecting the lungs emerging in late 2019. The highly infectious nature of SARS-CoV-2 resulted in an exponential increase in the number of patients hospitalised with acute respiratory disease [1]. This resulted in unprecedented health care demand which outstripped supply, particularly for supplemental oxygen and airway pressure support. The situation was particularly alarming in countries with limited resources like Bangladesh, where there were more than two million confirmed coronavirus disease 2019 (COVID-19) cases and 29 440 (1.45%) deaths by 31 December 2022 [2]. Mechanical ventilation in countries with developing health systems is a scarce resource, with poor availability and limited capacity to expand to meet needs as was seen in developed health care systems. Recent evidence has emerged that adult intensive care patients with COVID-19 required high oxygen supplementation [3] and benefitted from Positive End Expiratory Pressure (PEEP) [4]. The World Health Organization (WHO) however recommend the use of high-flow nasal cannula (HFNC) oxygen therapy over continuous positive airway pressure (CPAP) because of a lack of high-quality evidence in favor of CPAP [5]. HFNC oxygen therapy in adults is expensive, and its availability is somewhat limited even in the tertiary hospitals of Bangladesh.

A previous trial demonstrated that a locally made low-cost bubble CPAP was effective in significantly reducing mortality in children with severe pneumonia and hypoxaemia [6]. This led to the approval of this technology for the treatment of children with severe pneumonia and hypoxaemia by the Directorate General of Drug Administration (DGDA), Government of the People’s Republic of Bangladesh. During the early phase of the COVID-19 pandemic, a lack of appropriate respiratory support in health care facilities at all levels in Bangladesh resulted in a high case-fatality rates [7]. To address this problem a feasibility study was planned by icddr,b researchers in collaboration with The National Institute of Health Research (NIHR) Global Health Research Unit on Respiratory Health (RESPIRE), UK and Bangladesh University of Engineering and Technology (BUET) using an adaptive version of adult bubble CPAP device. We sourced a replacement of conventional plastic nasal interface by using a silicon based nasal interface that would provide a comfortable nostril seal in adult patients (Annex 1 in the **Online Supplementary Document**). Bioengineers from BUET provided a silicon-based 3D printed nasal interface (Annex 1 in the **Online Supplementary Document**).

Prior to the feasibility study, the investigators internally (with the approval of icddr,b-IRB) had accomplished the design testing phase of the study (from October to November 2020) in five healthy adults and initial safety phase (from November 2020 to April 2021) in six COVID and six non-COVID adult patients with severe pneumonia and hypoxaemia and/or acute respiratory distress syndrome (ARDS).

The design testing phase demonstrated that an adaptive version of bubble CPAP using a silicone based adult nasal seals could deliver effective PEEP (using digital manometer, model 82 152; 15 pounds per square inch (psi) with a mean pressure that was overall within +/−12% of target pressure, with most loss only at the highest pressure setting (15 cmH_2_O). The device was well tolerated by participants with normal vitals and no adverse events (File 1 in the **Online Supplementary Document**).

The initial safety phase revealed that the adaptive version of locally made bubble CPAP oxygen therapy in treating adults (COVID-19 positive and negative) with severe pneumonia and hypoxaemia was well tolerated without any adverse events. None of the study patients showed any features of carbon dioxide (CO_2_) retention. The saturation of peripheral oxygen (SpO_2_) of those patients settled (remained >93%) at 10 litres per minute (l/min) oxygen flow and 10 centimetres (cm) of water pressure PEEP. Vital signs were within normal physiological limits and all of them survived.

As a prelude to a future randomised controlled trial, we designed this feasibility phase of the study, aiming to further understand safety and acceptability, and also the operational challenges of introducing this technology to adult patients with severe pneumonia and hypoxaemia. Adapting this technology, if feasible and scaled up, could potentially reduce the need for mechanical ventilation and subsequently averting deaths among adult COVID-19 patients.

## METHODS

The study comprised two components: 1) a cohort safety study of adult patients with severe pneumonia and hypoxaemia receiving bubble continuous positive airway pressure (bCPAP) and 2) a qualitative evaluation of the barriers and operational challenges related to the introduction of bCPAP in an adult acute care setting. Feasibility was assessed within both components. The study endpoints were to assess a) the proportion of COVID-19 positive or negative adults with severe pneumonia and hypoxaemia, b) safety, including treatment failure and adverse events, c) ease of use and acceptability of bubble CPAP oxygen therapy by qualitative assessment.

### Study settings

We conducted this study in two tertiary-level hospitals in Dhaka, namely Dhaka Hospital of International Centre for Diarrhoeal Disease Research, Bangladesh (icddr,b) and Dhaka Medical College Hospital (DMCH). There was one makeshift intensive care unit (ICU) in the icddr,b Dhaka Hospital and an inpatient ward including ICU in DMCH. In these facilities, WHO standard low-flow (LF) oxygen therapy is the standard treatment for adult patients with severe pneumonia and hypoxaemia. Intravenous (IV) antibiotics, maintenance hydration/fluid, and other supportive care were also administered to the patients.

### Study design

#### Clinical study

We conducted a descriptive assessment of clinical and safety outcomes of study adults with severe pneumonia with hypoxaemia. Safety was evaluated by monitoring of adverse events (including bleeding, obstruction, breathlessness, pneumothorax, pneumomediastinum, abdominal distension) and treatment failure rate and deaths. Participants were adult patients aged between 18-64 years with severe pneumonia and hypoxaemia (SpO_2_<90% but ≥80% on room air) or ARDS with or without reverse transcription polymerase chain reaction (RT-PCR) positive for COVID-19. Severe pneumonia was defined as the presence of either radiological evidence of pneumonia and/or hypoxaemia, in patients presenting with cough, fever, respiratory difficulty and/or respiratory rate >30/min. ARDS was defined as SpO_2_/fraction of inspired oxygen (FiO_2_)≤300 millimetres of mercury (mm Hg). Potential participants were excluded, if they had severe hypoxaemia (SpO_2_<80% in room air) or had the presence of severe co-morbidities. Participants were provided with a study information leaflet and written informed consent was obtained. Enrolment commenced on 8 October 2021, at icddr,b and DMCH and was completed on 17 February 2022. Prior to the commencement of the trial, prototype bCPAP devices were developed and tested in healthy volunteers (File 1 in the **Online Supplementary Document**).

The diagnosis of severe pneumonia and hypoxaemia was done by the hospital physicians following national guidelines (Annex 2 in the **Online Supplementary Document**) [1]. After confirmation of the diagnosis and subsequent written informed consent, adaptive bCPAP was introduced immediately with 10 l/min oxygen flow and 10 cm of PEEP. Clinical study observations were made at baseline, then one and four hours after the start of adaptive bCPAP oxygen therapy and also at discharge. Further interim regular clinical observations were recorded by attending hospital physicians and nurses. Treatment was provided according to the National Guidelines on Clinical Management of Coronavirus disease 2020 (Annex 2 in the **Online Supplementary Document**) [1]. Site staff were trained and provided with personal protective equipment to enable appropriate handling of patients with COVID-19. Site investigators reviewed the clinical record forms (CRFs) within 24 hours of completion. Prior to the initiation of screening and enrolment, a training session for the hospital physicians and nurses at both sites was organised by the study staff. Safety was assessed by adverse event related to any of the study interventions or treatment failure during the hospital course, monitored and documented in the study CRF. Treatment failure of bCPAP was considered to have occurred if SpO_2_ remained <90% or fell <90% with respiratory difficulty after one hour of intervention (Annex 2 in the **Online Supplementary Document**).

Chest x-rays (CXRs) were performed and scored by a qualified radiologist independent from the study. The purpose of the scoring system was to detect the severity of COVID-19 infection based on the involvement of the consolidation or ground glass opacities (GGO) seen in the chest x-rays. Depending on the extent of involvement by consolidation or GGOs (0 = no involvement, 1 = <25%, 2 = 25-50%, 3 = 50-75% and 4 = >75% involvement), for each lung, with a maximum score of 8 for CXR. The scoring method was followed according to Marcelo et al. and Wong et al. [8,9].

#### Qualitative evaluation

We recruited two cohort groups. First, medical and nursing study-trained staff who provided care to adults with severe pneumonia and hypoxaemia admitted to the participating hospitals. The second group were adult patients who had severe pneumonia with hypoxaemia and were eligible and received bCPAP oxygen therapy. All participants provided written informed consent. We used a descriptive phenomenological approach [10,11] to examine operational challenges and opportunities relating to the implementation of bCPAP oxygen therapy in two Bangladeshi tertiary hospitals. This study was conducted in a participatory approach, with all stakeholders involved in bCPAP oxygen therapy, including consumers or beneficiaries (patients/attendants) and hospital clinicians – service providers (physicians and nurses). This integrated method aimed to fill knowledge gaps and provide multiple perspectives on complex, contextual, and multi-dimensional phenomena.

The qualitative evaluation was supervised by a qualified and experienced anthropologist (MFU), and assisted by two Research Assistants (RAs). With agreement with study principal and other investigators they purposefully [10,12] selected 16 participants (patient’s attendants, n = 12, and clinicians, n = 4) from Dhaka Hospital, icddr,b and 15 participants (parents/other family members, n = 9, and clinicians, n = 6) from DMCH to conduct 31 in-depth interviews (IDIs) and one focus group discussion (FGD) with eight participants (**Table 1**). These participants were closely involved in the care of patients receiving adaptive bCPAP. They selected different caregivers and influential family members based on their involvement in patient care, education level, occupation and patient age groups. We purposively selected clinicians who had worked in the same department for at least six months. Interviewers approached the participants in person to discuss the project and arrange interviews and FGDs at convenient time and place for the participants in the studied hospitals, followed by criteria of COREQ checklist (Additional file 2 in the **Online Supplementary Document**). Researchers had no relationship with participants prior to study commencement, and participants were not familiar with researcher or interviewers. No one approached declined participation. Only the interviewee and interviewer were present at the time of the study. Researcher conducted audio-recorded interviews and FGDs using an interview and FGDs topic guide. Short notes were taken by the interviewers during interview or FGDs to facilitate in-depth data collection (e.g. to ask relevant questions to the participants).

**Table 1:** Methods and study participants

Methods	Before introduction of bCPAP	After introduction of bCPAP
In-depth interviews (IDIs)	4 IDIs with hospital clinicians	6 IDIs with clinicians
	-	21 IDIs with caregivers
Focus Group Discussion (FGD)	1 FGD with 8 hospital clinicians	-
	In total = 12 participants	In total = 27 participants
	Total number of participants = 39	

bCPAP – bubble continuous positive airway pressure

Interviewers (RAs) had a social science academic background and were experienced in collecting qualitative data locally. They were trained by a qualified anthropologist (MFU). They interviewed clinicians in hospitals at a place and time that were convenient for the participants. After receiving bCPAP oxygen therapy and being discharged from study hospitals, interviewers conducted IDIs with patients' caregivers in their households. The average duration of an interview was 47 minutes. We used flexible semi-structured data collection guidelines and recorded by audio-recorders with permission. Based on the study objectives, interview guidelines were developed and pilot-tested. They continued collecting data until reaching data saturation.

#### Data analysis

Clinical safety study: descriptive analyses were performed on baseline characteristics and study outcomes. We calculated the proportion of COVID-19 positive or negative adults with severe pneumonia and hypoxaemia, treated with adaptive bCPAP oxygen therapy in tertiary-level hospitals of Bangladesh. The analysed safety outcomes included adverse events, treatment failure rate and deaths of COVID-19 positive and COVID-19 negative adults with severe pneumonia and hypoxaemia and/or ARDS were described.

Qualitative data: recorded interviews and FGDs were transcribed verbatim. The lead anthropologist (MFU) first developed a deductive code list based on study objective and interviews or FGD guide and then carefully selected four transcriptions with rich information and carefully read and coded them with the help of two RAs to develop inductive code list (emerged new codes from empirical data). Finally, the lead anthropologist compiled the deductive and inductive code lists to develop a final code list for manually coding all transcribed data using Microsoft word. The lead anthropologist and two RAs coded each transcript, compared the results, and resolved any discrepancies to ensure the trustworthiness of the coding process. We used a thematic coding approach to identify main themes for narrative interpretation of the data [10,13]. For analysing data, we followed a sequence of steps [13] that included reading, coding, re-reading, displaying data, reducing, and interpreting textual data using an emic approach [14] after that the main themes were finalised.

#### Ethical and governance approvals

The study was approved by the Research Review Committee and Ethical Review Committee of icddr,b (PR-21051) and the Ethics committee of the University of Edinburgh and ACCORD governance. We also obtained administrative approval from the Ministry of Health & Family Welfare, Bangladesh for conducting this study.

## RESULTS

**Figure 1:**
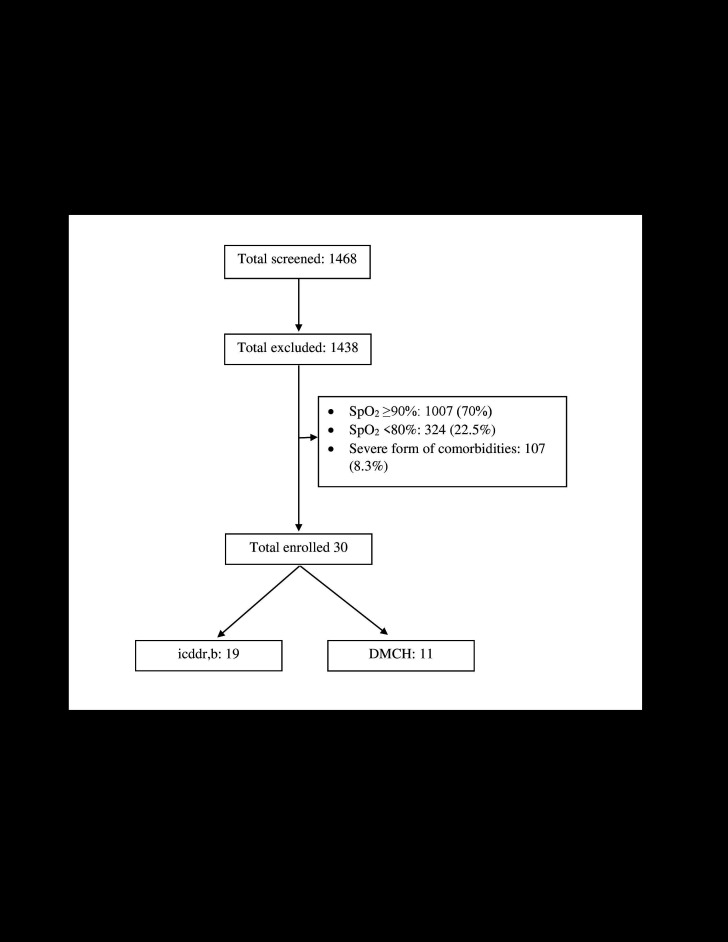
Study outline.

Clinical study: a total of 1468 patients were screened. Among them, 1007 (70%) patients did not have hypoxaemia (SpO_2_<90%), 324 (22.5%) patients had severe hypoxaemia (<80% on room air) and 107 (8.3%) had severe form of co-morbidities. Thus, according to protocol we were able to enroll 30 of them; 19 patients were enrolled at the Dhaka Hospital of icddr,b and 11 patients were enrolled at DMCH. Malignancy, acute ischemic heart disease, heart failure, extrapulmonary tuberculosis, and kidney disease with an indication of dialysis were common co-morbidities (**Figure 1**).

Our study patients had a median age of 52 years (IQR 40, 60) and male and female ratio was 1:1. COVID-19 disease was confirmed through RT-PCR test on nasopharyngeal swabs from the study participants. RT-PCR reports of 3 (10%) patients were positive for COVID-19. Twenty-three (76.7%) participants had a history or documented fever, 22 (73.3%) participants had a cough and all participants had respiratory difficulties during enrolment (**Table 2**). Chest x-ray findings and scores are shown in **Table 3**.

**Table 2:** Demographic and clinical information

Demographic and clinical information			
	**All (n = 30)**	**icddr,b (n = 19)**	**DMCH (n = 11)**
Male	15 (50)	9 (47)	6 (55)
Age, median (IQR)	52 (40-60)	54 (45-64)	50 (35-58)
Fever	23 (76.7)	15 (78.9)	8 (72.7)
Cough	22 (73.3)	14 (73.7)	8 (72.7)
COVID-19 infection confirmed by RT PCR	3 (10.0)	0 (0.0)	3 (27.3)

icddr,b – International Centre for Diarrhoeal Disease Research, Bangladesh, DMCH – Dhaka Medical College Hospital, IQR – interquartile range, RT PCR – reverse transcription polymerase chain reaction

**Table 3:** Chest x-ray findings of study participants receiving bCPAP oxygen therapy

Chest x-ray			
	**All (n = 30)**	**icddr,b (n = 19)**	**DMCH (n = 11)**
Lobar consolidation	10 (33.3)	7 (33.3)	3 (27.3)
Bilateral pneumonia	13 (43.3)	7 (43.3)	6 (54.5)
Fibrotic changes seen in right lower zone	1 (3.3)	1 (3.3)	0 (0.0)
Ill-defined opacity in right lower zone	3 (10.0)	3 (10.0)	0 (0.0)
Inhomogeneous opacity in right lower zone	1 (3.3)	1 (3.3)	0 (0.0)
Non specific change in both lower zone	2 (6.7)	0 (6.7)	2 (18.2)
Chest x-ray score	3 ± 1	3 ± 1	3 ± 1

bCPAP – bubble continuous positive airway pressure, icddr,b – International Centre for Diarrhoeal Disease Research, Bangladesh, DMCH – Dhaka Medical College Hospital

To assess clinical safety, we assessed treatment failure (SpO_2_<90%) one hour after commencing bCPAP) and adverse events. Baseline vitals were recorded during enrolment before the start of bCPAP oxygen therapy and again after one hour of bCPAP (**Table 4**). SpO_2_ improved after an hour of initiation of bCPAP oxygen therapy with 10 l/min oxygen flow and 10 cm of PEEP in all participants. None of the participants required an increase in oxygen flow and PEEP in the first hour of enrolment. As such treatment failure was not identified.

**Table 4:** Vitals of the participants

Clinical findings in the participants				
		**All (n = 30)**	**icddr,b (n = 19)**	**DMCH (n = 11)**
Temperature (mean, SD)	Baseline findings on room air	36.78 ± 0.97	37.03 ± 1.03	36.34 ± 0.69
	After 1 h with bCPAP oxygen therapy	36.69 ± 0.85	36.95 ± 0.80	36.25 ± 0.77
Heart rate (mean, SD)	Baseline findings on room air	97 ± 17	93 ± 15	104 ± 19
	After 1 h with bCPAP oxygen therapy	94 ± 17	91 ± 15	100 ± 18
Respiratory rate (mean, SD)	Baseline findings on room air	30 ± 3	29 ± 3	30 ± 3
	After 1 h with bCPAP oxygen therapy	27 ± 4	26 ± 4	28 ± 4
Systolic BP (mean, SD)	Baseline findings on room air	109 ± 16	104 ± 14	117 ± 17
	After 1 h with bCPAP oxygen therapy	111 ± 19	106 ± 14	121 ± 22
Diastolic BP (mean, SD)	Baseline findings on room air	72 ± 12	68 ± 6	80 ± 15
	After 1 h with bCPAP oxygen therapy	73 ± 13	68 ± 7	82 ± 15
SpO_2_ (mean, SD)	Baseline findings on room air	87 ± 2	87 ± 2	86 ± 2
	After 1 h with bCPAP oxygen therapy	98 ± 2	98 ± 2	98 ± 2

icddr,b – International Centre for Diarrhoeal Disease Research, Bangladesh, DMCH – Dhaka Medical College Hospital, SD – standard deviation, h – hour, bCPAP – bubble continuous positive airway pressure, BP – blood pressure

No adverse events including bleeding, obstruction, breathlessness, pneumothorax, pneumomediastinum, abdominal distension during and after the trial were reported. Two patients had minor bruises at ala of the nose and it completely subsided with topical ointment. Two patients were referred to another hospital as one had high-level troponin I and another had progressive acute kidney injury with oliguria from the baseline and we referred the patients after successful completion of intervention with bCPAP oxygen therapy. These adverse events were unrelated to the study intervention. The rest of the patients were discharged successfully. Thus, there was no deaths or serious adverse event.

The average duration of bCPAP oxygen therapy (stopped when SpO_2_>93% for >15 minutes in room air and respiratory rate <30/min) was 14.4 hours (±24.8) (**Table 5**). Twenty-seven (90%) participants required one episode or cycle of bCPAP oxygen therapy and their average duration of bCPAP oxygen therapy was 8.0 hours. Two (7%) participants required two episodes and one (3%) participant required three episodes of bCPAP oxygen therapy as they again developed hypoxaemia after initial resolution of hypoxaemia during the hospital stay and their average total duration of bCPAP oxygen therapy was 101.5 hours and 24 hours respectively.

**Table 5:** Outcomes of study participants receiving bCPAP oxygen therapy

Outcome			
	**All (n = 30)**	**icddr,b (n = 19)**	**DMCH (n = 11)**
Well and discharged	28 (93.3)	17 (89.5)	11 (100.0)
Died at hospital	0 (0)	0 (0)	0 (0)
Referred to superior facility	2 (6.7)	2 (10.5)	0 (0)
Left against medical advice	0 (0)	0 (0)	0 (0)
Withdrawn from the study by caregiver	0 (0)	0 (0)	0 (0)
Withdrawn from the study by physician	0 (0)	0 (0)	0 (0)
Duration of bCPAP oxygen therapy (mean, SD)	14.4 ± 24.8	14.7 ± 29.4	14.0 ± 15.1
Duration of hospital stay (median, IQR)	91 (66-215)	75 (50-91)	232 (148-350)

bCPAP – bubble continuous positive airway pressure, icddr,b – International Centre for Diarrhoeal Disease Research, Bangladesh, DMCH – Dhaka Medical College Hospital, SD – standard deviation, IQR – interquartile range

The adult adaptive version of bCPAP having a silicon-based 3D printed nasal seal was found to be comfortable and well tolerated by all the participants.

### Qualitative study findings

Both hospitals had central piped oxygen available as well as adequate and well-functioning oxygen cylinders, suction machines, power generator back-up, and bio-engineers for regular maintenance of medical equipment. However, limited pulse oximeter availability in DMCH required provision of study oximeters by the study team before the introduction of clinical use of adaptive bCPAP there. There was a high patient load (on average 40 patients admitted in the ward per day) and staff numbers, especially physicians and nurses, were inadequate (morning shift –

 three physicians, six nurses; evening shift – two physicians, three nurses; night shift – one physician, three nurses) particularly at night. This resulted in some missing clinical reviews/follow-ups of vitals parameters of hospitalised study patients. Patients in DMCH were often required to purchase medicines whereas medicines were free of cost in icddr,b. Other challenges and opportunities are shown in **Table 6**.

**Table 6:** Observation of operational challenges and opportunities during the implementation of bCPAP oxygen therapy

Themes	Dhaka Hospital of icddr,b	DMCH	Provided support to enable the feasibility study to go ahead
Lack of holder of water filled plastic bottle of adaptive bCPAP circuit	Due to lack of holder of the water filled plastic bottle, we kept the bottle on the tool/table beside the bed of the patient. As a result, we experienced occasional fall of the bottle on the ground resulting in cessation of bCPAP oxygen therapy transiently.		Attendant of the patient informed the nurses and they fixed the bottle with the circuit. However, in future intervention studies the holder needs to be attached to each bed by the hospital administration.
Auto bubbling before the introduction of nasal interface into the nostrils of the patient	Observed on two occasions. One observed just after initiation of oxygen therapy but before the fixation of nasal interface into the nostrils and one during receiving bCPAP oxygen therapy resulting from the collection of condensate in the expiratory arm of the bCPAP circuit.		Nurses/doctors identified the auto bubbling inside the water filled plastic bottle after initiation of oxygen, just prior to fixation of the nasal interface in to the nostrils of the patient and thus changed the circuit. However, accumulation of condensate was identified by the attendant who informed the nurses and the nurse changed the circuit.
Lack of adequate bubbling in the water filled plastic bottle through the expiratory arm of bCPAP	Infrequently observed, especially due to mouth breathing during sleeping; loose fitting of seal in nasal interface; and kinking of the tube anywhere in the circuit		The attendant of the patient was taught at bedside and they were able to inform nurses in case of lack of bubbling. The nurses fixed them to reproduce the bubble.
Discomfort of female patient with nose pin	Two female patients with nose pins had faced difficulty during the fixation of nasal interface in to nostrils as they refused to open the nose pin		Nurses tried to fix them with relatively smaller nasal interface to minimise the discomfort.
Experience of attending doctors and nurses	Routine follow-ups of vitals of the patients were missed especially at night due to high patient load and lack of sufficient number of clinicians and at the later stage of the study due to rapid turn-over of trained staff. Adaptive version of adult bCPAP was found to be simple to handle; no adverse events; rapid disappearance of respiratory distress and rapid recovery; reduced bed occupancy, clinicians’ workload; ultimately reduced hospital cost per patient		If there was missing follow-up in time especially at night, the attendant of the patient was advised to ask the nurse on duty to accomplish the follow-ups (this approach worked occasionally).
Experiences of parents/other family members of the patients	Some patients and their attendants had initial anxiety of this new intervention; once the patients had comfort in breathing, they were very relaxed. They were also happy for hospital stay that helped to reduce parents/other family members direct and indirect cost of purchasing food, medicines and traveling to the hospitals.		Reassured

bCPAP – bubble continuous positive airway pressure, icddr,b – International Centre for Diarrhoeal Disease Research, Bangladesh, DMCH – Dhaka Medical College Hospital

## DISCUSSION

The main observation of our study was that the adapted version of bCPAP used to treat adults with severe pneumonia and hypoxaemia with or without ARDS was safe, well tolerated and not associated with treatment failure across all study participants.

The median age the patients receiving adaptive bCPAP was 52 years with mean arterial oxygen saturation 87% in room air. It is important to note that the IRB obliged us not to enroll the patients beyond age of 64 years and 80% saturation with severe co-morbidity as they felt that the new intervention required to have some safeguard to minimise potential harms. This is one of the limitations of our study and we need to include the population beyond age of 64 years and <80% saturation with severe co-morbidity in our next randomised trial. The mean duration of bCPAP oxygen therapy was around half a day and there were no treatment failure or deaths or any serious adverse events related to adaptive bCPAP oxygen therapy. These observations support the overall good impression of attending physicians and nurses about adaptive bCPAP and increase the confidence level of the investigators to carry forward with this intervention in to the next stage for the treatment of more severe hypoxaemic patients. An RCT would be required to understand the efficacy of adaptive bCPAP oxygen therapy compared to WHO standard low flow oxygen therapy for the treatment of COVID-19 positive or negative adults with severe pneumonia and hypoxaemia and/or ARDS.

Another important observation is the qualitative evaluation of the barriers and operational challenges related to the introduction of bCPAP in an adult acute care setting. The main challenges included the lack of adequate numbers of doctors and nurses in the ward leading to failure of routine review, especially overnight for some patients receiving adaptive bCPAP. This was exacerbated by the rapid turnover of the trained indoor physicians and nurses. To minimise these impacts motivational meetings were provided to the study investigators, hospital doctors, nurses and administrators. A WhatsApp group of clinicians for the bCPAP feasibility study was also created where all enrolled patients were reviewed by the study investigators, to minimise the potential impact limited night reviews. There remained a concern at the very high frequency of in-person training provided to the clinicians, to ensure the rapid turnover did not impact patient safety during the study, and how feasible this might be in any future trial and implementation.

Fixation of the water filled plastic bottles upright was also problematic. Temporary solutions were introduced (bedside tables), however a better solution will be sought prior to any potential RCT. Auto bubbling was observed in few cases especially if there was any resistance in the nasal interface or if the nostrils were blocked with nasal secretion. To eliminate the first reason, nurses were requested to look for the auto bubbling inside the water filled plastic bottle after fixing the proximal arm with oxygen cylinder and distal arm with water filled plastic bottle, just prior to fixation of the nasal interface in to the nostrils of the patient. We trained the attendant to be alert regarding the accumulation of nasal secretions or condensate in the distal arm of the bCPAP circuit, so that they could inform the nurses. In all the instances our nurses changed the circuit to fix the problem. Lack of bubbling mainly occurred due to kinking or leaking of the any part of cannula of adaptive bCPAP circuit or due to loosening of the nasal interface.

Our study identified that oxygen availability was not an issue as both hospitals had good reserves of oxygen, using both central piped oxygen and back-up cylinders. This was possible potentially due to the advance readiness of oxygen security in two leading hospitals in the country. DMCH medicine ward did not have a functioning pulse oximeter (which is the practical scenario at almost all public hospitals in the country) and so was supplied with a pulse oximeter and training by the study team.

The result of the study indicates that most of the challenges identified during the feasibility of adaptive bCPAP in treating adults with severe pneumonia and hypoxaemia and/or ARDS are addressable. The result further demonstrates that after addressing the operational challenges, adaptive bCPAP oxygen therapy was found to be feasible for the treatment of severe pneumonia with hypoxaemia and/or ARDS irrespective of COVID-19 positivity status. The patients, their attendants and medical staff were satisfied with this innovative treatment method. Their perception was that it helped rapid improvement and reduced oxygen requirement for the patient. None of the patients required a high flow nasal cannula or mechanical ventilation which eventually reduced ICU admission. These could ultimately result in reducing the hospital treatment costs.

Adaptive bCPAP would be feasible to be tested for an efficacy trial (adaptive bCPAP vs LF oxygen therapy) in tertiary hospitals in Bangladesh if the hospitals are supplied with well-functioning good quality pulse oximeters and enhanced training of the doctors and nurses and bedside motivational education of the attendant of the patients. Were efficacy trial results favorable then national introduction would also require testing in district general hospitals via an effectiveness trial followed by implementation research. Thus, accomplishment of these steps may help for advance readiness of potential future crisis of improved respiratory support especially in low resource settings.

## CONCLUSIONS

Our study demonstrated some addressable operational challenges during the clinical use of adaptive version of bCPAP oxygen therapy in tertiary hospitals in Bangladesh. Our study further demonstrates that the clinical use of locally made low cost adaptive bCPAP oxygen therapy appears safe, is feasible and warrants an efficacy trial to understand its beneficial role over WHO standard LF oxygen therapy.

## Additional material


Online Supplementary Document
