# Supplementary material for: Evaluation of feasibility phase of adaptive version of locally made bubble continuous positive airway pressure oxygen therapy for the treatment of COVID-19 positive and negative adults with severe pneumonia and hypoxaemia
Source: J Glob Health. 2023 Nov 24;13:06046. doi: 10.7189/jogh.13.06046 (PMC10668204; doi:10.7189/jogh.13.06046)
Supplement: Online Supplementary Document [file jogh-13-06046-s001.pdf]

Supplemental Information

Annex 1.

I. Change/version history

S. No	Version No.:	Effective date	Changes /comments
1	1.0	01/11/2020	New

II. Distribution

**S. No**	**Unit**
1	Dhaka Hospital, icddr,b
2	Dhaka Medical College Hospital

III. Issue date for current version: 01-11-2020

1. Objective
   This SOP is intended to guide study personnel to properly assemble locally developed bubble CPAP.

2. Applicability to and responsibilities of various staff members

**Staff member**	**Responsibility**
Study physician	• Ensure availability of all the necessary materials
Site supervisor/Paediatrician	• Supervise proper assembly & administration of bubble CPAP to trial participants
Paediatrician/ Medical Officer and Study Nurse	• Assemble bubble CPAP using the necessary equipments
Study Nurse	• Administer oxygen using bubble CPAP
Paediatrician/GP and Study Nurse	• First one hour of admission: Close monitoring of the patient, oxygen flow and bubble CPAP device<br>• There will be 4 hourly follow-ups of study patients after a 1st hour follow-up that include monitoring of the patient, oxygen flow and bubble CPAP device
Study Nurse	• Cleaning of all the connecting parts of bubble CPAP with chlorinated water every 3 or 4 day interval
PI/Study physician	• Refresher training every 6 months interval

3. Required materials and Equipment

Item	Number	Specification
Adaptive version of nasal canula	1	Adult size (universal)
Nasal prong/canula	1	Adult size (universal)
IV infusion set	1	None
Graduated water bottle	1	None
Oxygen cylinder	1	None
Hand gloves	1	Disposable
Mask	1	Disposable, N95

4. Step-by-step procedures

4.1 Cut one of the limbs of the nasal prong and tie up the distal end.
4.2 Connect proximal limb of the nasal prong to IV fluid tubing.
4.3 Insert the distal end of the IV fluid tubing to a graduated water bottle.
4.4 Make the depth of the inserted tube into the water at 10 cm. which can be increased up to 15 cm. depending on the patient’s response.
4.5 Connect the main distal limb of the nasal prong will to oxygen source/ concentrator.
4.6 Check for auto bubbling

N.B Auto bubbling means if the bubbles are produced after initiation of oxygen but before fixing the nasal interface into the nostrils.
If auto bubbling occurs, change the whole circuit and reconstruct again.

4.7 Connect the nasal prong to the nasal seal and securely insert to the nostrils of the patient.

5. Safety measure

The whole device or circuit is hazardous neither to patients nor to the clinical staff. Before the preparation the individual needs to wear gloves. Follow-up every 4 hourly to observe for any obstruction/disconnection/leakage, bubbling, condensate in the expiratory arm in bubble CPAP circuit, nasal trauma/bleeding, abdominal distension or air leak syndrome (pneumopericarium, pneumomediastinum). BCPAP bottle and connecting parts can be reused by cleaning with chlorinated water properly. The BCPAP bottle and nasal seal will be submerged in 70% isopropyl alcohol for 72 hours before use.

The perforation and water pressure (in centimeter) marking will be done at the procurement of icddr,b. All the BCPAP bottles were supplied from icddr,b Dhaka Hospital procurement. The bottles were kept in medical equipment containing box or shelf away from sunlight to avoid frequent handling. This devices were not reused for many times.

6. Quality Assurance / Quality Control

All the physicians and study nurses will be trained in this SOP. Refresher trainings will be conducted after every six months for all study staff. The site supervisor also oversees the study personnel when they assemble bubble CPAP.

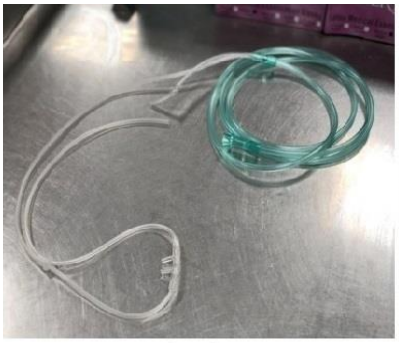

Nasal Canula

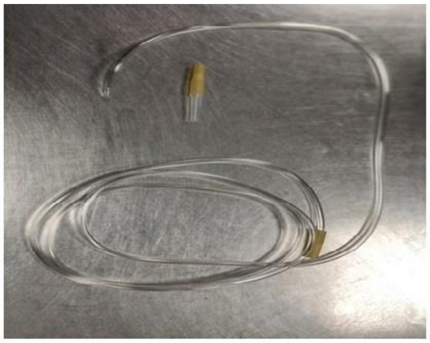

Infusion set

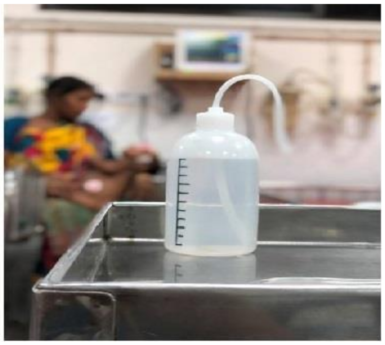

Bottle

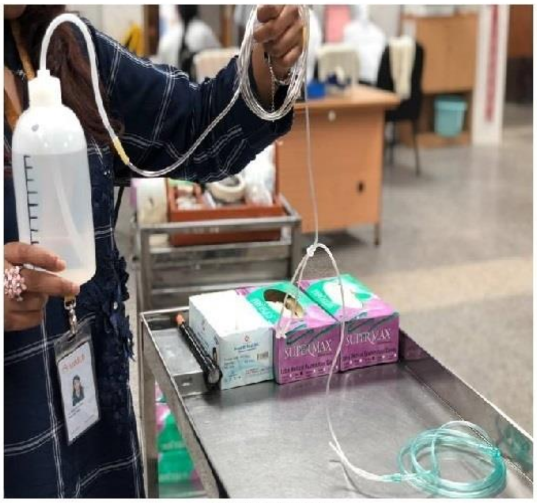

Prepared circuit

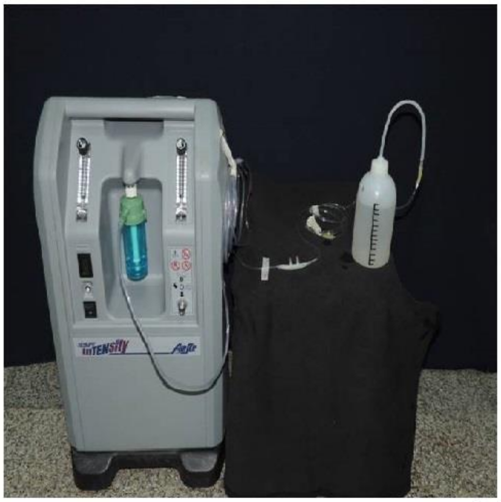

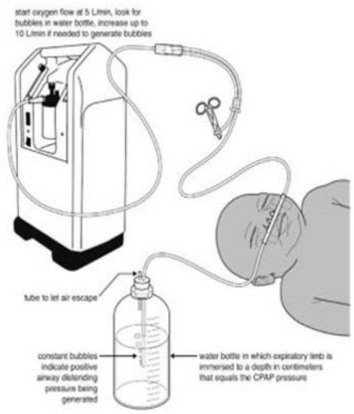

Bubble CPAP

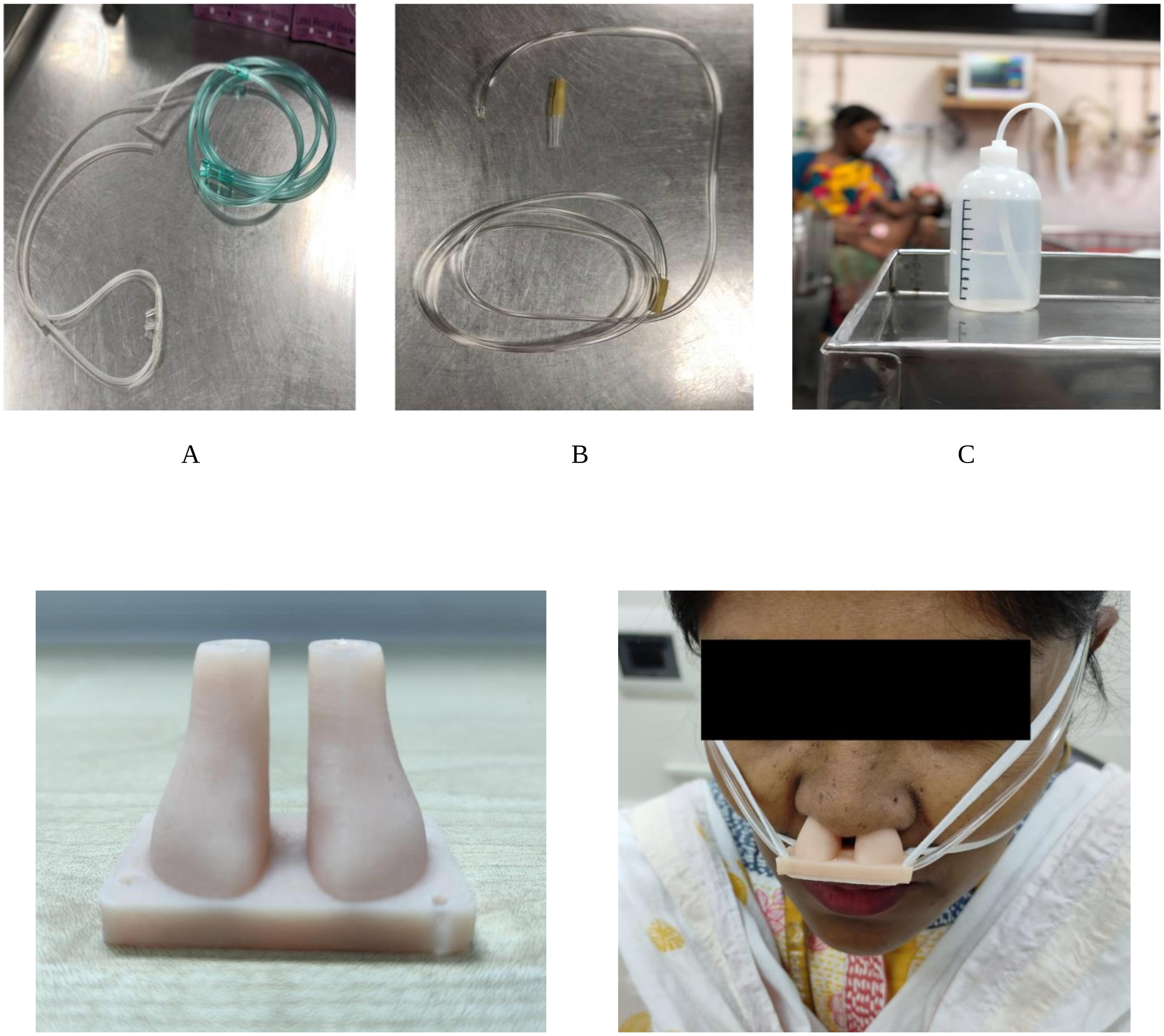

Fig: (A) Nasal cannula, (B) Infusion set, (C) BCPAP bottle, (D) Nasal seal, and (E) Use of nasal seal

Supplemental Information

Annex -2

Diagnosis and management of COVID-19 patients according to the National Guidelines on Clinical Management of Coronavirus disease 2020

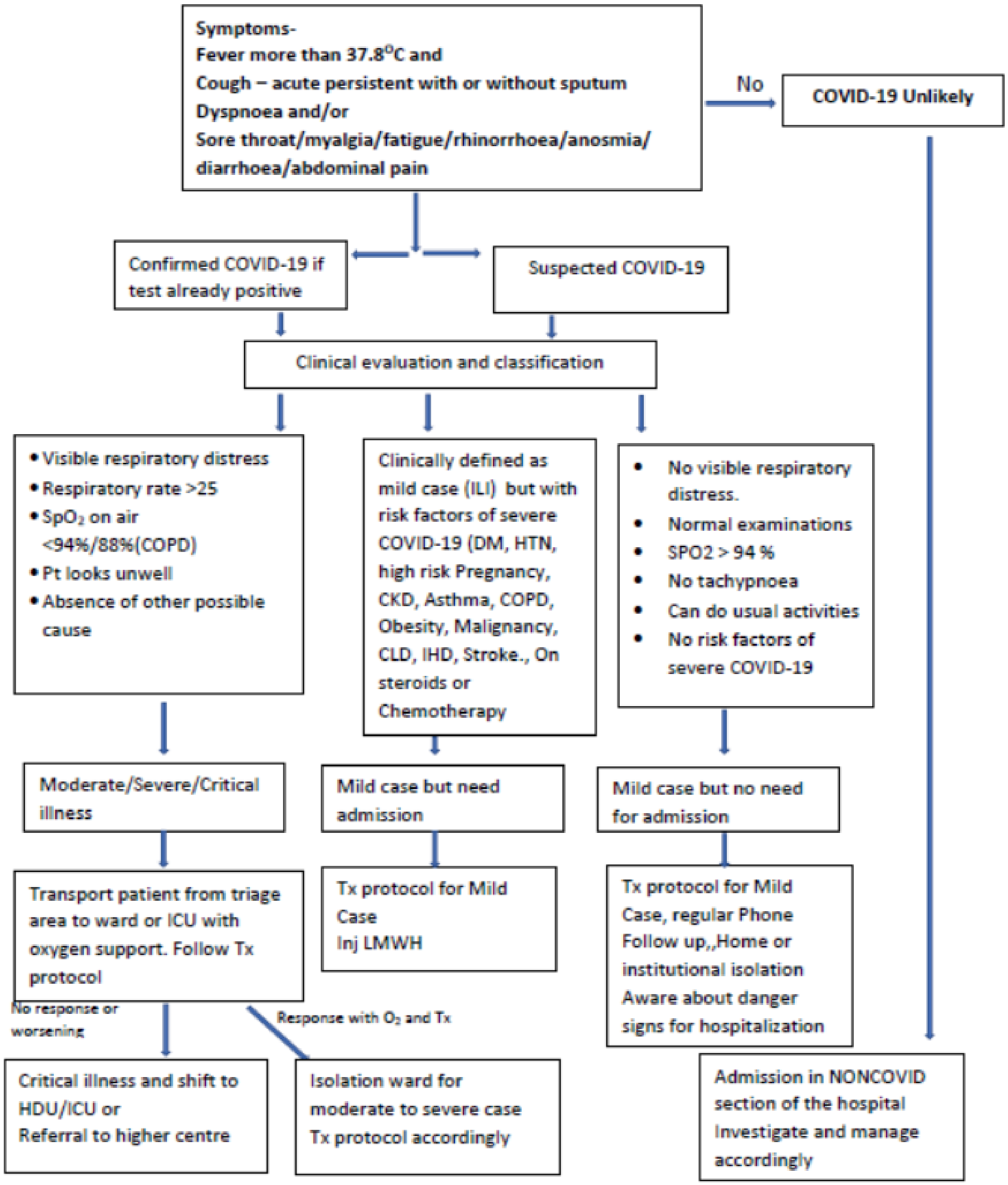

This is a simplified triage for every hospital in Bangladesh (Public or private).

Clinical Classification

For the practical purpose of patient management, the six syndromes of COVID-19 have been categorized into mild, moderate, severe and critical cases.

Clinical classification for case management		
01	Mild	Influenza like illness (ILI)
02	Moderate	Pneumonia (CRB 65 score 0)
03	Severe	Severe Pneumonia, Sepsis
04	Critical	ARDS, Septic shock

N.B. Some patients of COVID-19 may have hypoxia without clinically evident dyspnoea and cyanosis. Thus, wherever available, pulse oximetry should be used to rule out hypoxia and respiratory failure.

N.B. After evaluation and testing for respiratory distress, cases due to cardiac causes/ non COVID-19 causes will be sent to NON COVID zone.

Clinical Case definition

1. Mild cases

- The clinical symptoms are mild, and there is no sign of pneumonia on imaging.
- Symptoms may be: fever, cough, sore throat, malaise, headache, muscle pain without shortness of breath or abnormal imaging

2. Moderate cases

- Adolescent or adult with clinical signs of pneumonia (fever, cough, dyspnoea, fast breathing) but no signs of severe pneumonia.
- Respiratory distress with < 30 breaths /min
- Pulse oxymetry showing saturation > 90% at ambient air

3. Severe cases

Cases meeting any of the following criteria:

- Respiratory distress (≧30 breaths/ min);
- Finger oxygen saturation ≤90% at rest;
- Arterial partial pressure of oxygen (PaO_2)/fraction of inspired oxygen (FiO_2)≦300mmHg (1mmHg=0.133kPa)

4. Critical cases

Cases meeting any of the following criteria:

- Respiratory failure and requiring mechanical ventilation.
- Shock.
- With other organ failure that requires ICU care.

D. Severe cases with respiratory symptoms

Management of Moderate case protocol (Except oral steroid)

Plus

- Steroids- **Inj Dexamethasone 6 mg daily for 10 days**

  or

  Inj Methylprednilone-250 mg daily for 5 days (switch to IV from oral if already started)
- Maintain euvolaemia (Avoid fluid load)
- Early Norepinephrine for hypotension
- **Broad spectrum antibiotics— IV drug at the discretion of consultant**
- Consider for cytokine storm/ HLH (Hemophagocytic lymphohistiocytosis) picture:
  1. Tocilizumab*
  2. Convalescent Plasma therapy**

*Tocilizumab: Adult Dosing (>18 years): 8 mg/kg (max 800 mg/dose) • Pediatric Dosing (<18 [illegible]

Indication of Tocilizumab:

1. COVID-19 positive and All of the following respiratory findings:
   a. Abnormal chest imaging consistent with COVID-19
   b. Rapidly worsening gas exchange/respiratory status over 24-48 hours and requiring >6 L/min O2 or on mechanical ventilation
2. Absence of systemic bacterial, fungal or parasitic co-infection
3. High clinical suspicion for cytokine release syndrome and clinically deterioration of the patient

Convalescent Plasma therapy: Donor should be healthy subject recovered from COVID-19 and preferably after 28 days with neutralizing titre more than 1:160 and binding titre more than 1:1000

Indication of convalescent plasma:

1. Age >18 years old
2. Positive SARS-CoV-2 and symptoms more than 8-10 days
3. Informed consent
4. Severe or life-threatening disease defined by at least one of the following:
   - Increasing dyspnea
   - Respiratory rate >30
   - SpO2 <88%
   - P/F ratio <300
   - Lung infiltrate >50% within 24-48 hours
   - Septic shock
   - Multi organ failure

N.B:

1. In severe case, broad spectrum antibiotic can be prescribed if secondary bacterial infection is suspected and the choice of drug should be under the discretion of consultant working in hospital.

2. In severe case, If Antiviral drug (IV Remdesivir) is prescribed, it is better to prescribe it as early as possible.

3. In severe cases, after appropriate therapy with Oxygen, antiviral and steroids, if there is clinical deterioration and there is indication of IL-6 inhibitor and or convalescent plasma present, then these modalities of treatment should be provided with frequent monitoring of disease and drug related phenomena.

4. Prior to administration of convalescent plasma, the binding titer and or neutralizing titer should be measured in donor from appropriate centre and expert opinion should be sought.

6. Immunomodulator, Monoclonal antibody or JAK inhibitor should not be used without evidence of clinical trial

Thromboprophylaxis in severe case:

- LMW heparin (Inj enoxaparin) enoxaparin 1mg/kg/day SC twice daily (dose adjust with CrCl< 30ml/min)

  Or if LMWH cant be given or contraindicated

  Inj unfractionated heparin (UFH): 60U/kg bolus+12units/kg/hr infusion-for ACS

  80U/Kg bolus +18units/kg/hr infusion-for VTE and AF
- Thromboprophylaxis should be given until symptom resolves or improves and followed by tab rivaroxaban 10 mg once daily for 2 months
- Appropriate dose (upper limit) of anticoagulant should be practised in severe to critical illness of COVID-19

E. Critical cases with respiratory symptoms (SOB; hypoxia: admit to ICU)

In addition to the treatment protocol stated above, following measures are part of critical case management.

Escalation of respiratory support: Following are important for severe to critical patients who need intensive monitoring:

- **Prone positioning (more than 12 hrs/day) for improvement of Oxygenation.**
- **Low flow O2 delivery devices:**
  Nasal cannula (up to 4-6LPM and provide up to 50% FiO2; Simple mask (up to 10 LPM and provide up to 60% FiO2); Venturi mask (up to 15 LPM and provide 24 -60% FiO2); Partial rebreather mask (15 LPM and provide 70% FiO2); Non rebreather mask (15 LPM and provide 100% FiO2)
- **High flow delivery device:**
  High Flow Nasal cannula (HFNC): up to 60-70 LPM and provide 100% FiO2
  Advantages: Well tolerated, generate PEEP in closing mouth.
- **Non-invasive positive pressure ventilation:**
  CPAP: (Setting 5-20 cmH2O) and used for type I respiratory failure;
  BiPAP (Setting EPAP 4-16 cm H2O, IPAP 10-20 cmH2O and minimum pressure support 4 cmH2O) and used for both type I and type II respiratory failure.
  Increase CPAP or EPAP for hypoxia
  Increase pressure support (IPAP-EPAP) for hypercapnia
- **Mechanical ventilation.** (When all the above measures failor indicated.)

Monitoring for severe to critical patients (if facility is available)

Depends on the severity of the patients and discretion of the consultant. To close monitor of the vital organs following investigations can be recommended

- Daily: CBC, CRP, RBS, ECG, Ferritin, , D-dimer, Electrolyte. Blood urea , Serum Creatinine, LFT , ABG(if possible) CRP and Ferritin are good biomarkers and track disease severity.
- Follow CXR, CT scan of Chest or Bed side USG
- Follow Tropinin and ECHO closely; patient sometime develops severe cardiomyopathy/myocarditis.

Hospital care Principles

- Mild cases with risk factors, severe and critical cases of suspected (or probable) or confirmed COVID-19 require hospital care.
- Management of such patients warrant immediate implementation of appropriate infection prevention and control measures.
- Patients with severe disease often need oxygenation support.

- Aerosol generating procedures such as endotracheal intubation, bronchoscopy, nebulization, cardiopulmonary resuscitation, open suctioning of respiratory tract, tracheostomy etc. demand specific protection of healthcare workers with appropriate personal protective equipment (PPE).
- The safety of high-flow oxygen and non-invasive positive pressure ventilation in these measures is uncertain, and they should be considered aerosol-generating procedures that warrant specific isolation precautions.
- Patient with sepsis with or without shock may require treatment in high dependency unit (HDU) or ICU depending on disease severity and clinical judgement of treating physicians.
- If patients develop acute respiratory distress syndrome, intubation with mechanical ventilation will be needed.

- Aerosol generating procedures such as endotracheal intubation, bronchoscopy, nebulization, cardiopulmonary resuscitation, open suctioning of respiratory tract, tracheostomy etc. demand specific protection of healthcare workers with appropriate personal protective equipment (PPE).
- The safety of high-flow oxygen and non-invasive positive pressure ventilation in these measures is uncertain, and they should be considered aerosol-generating procedures that warrant specific isolation precautions.
- Patient with sepsis with or without shock may require treatment in high dependency unit (HDU) or ICU depending on disease severity and clinical judgement of treating physicians.
- If patients develop acute respiratory distress syndrome, intubation with mechanical ventilation will be needed.

Supplemental Information

Additional File 1. Design phase

Development of adaptive version of paediatric bCPAP for delivery to adult participants:

To prevent nasal leaking, we have developed a prototype nasal cannula that is silicon-based and ergonomically designed considering the varying size and direction of adult nasal cavities as well as the comfort of patients (in collaboration with colleagues at Biomedical Engineering department of Bangladesh University of Engineering and Technology (BUET). The silicone devices are produced and designed in a 3D Computer-Aided Design (CAD) software at BUET. The prototypes are initially tested by 3D printing. Finally, a mold is designed and printed using 3D printing. The mold is filled with the liquid medical-grade silicone and dried. It is cost effective and can be produced at any time.

We used oxygen cylinders for the whole study duration as the central oxygen supply facility is not available in most hospitals in Bangladesh, thus to make it affordable and sustainable we used oxygen cylinders.

Transparent plastic bottles: A bigger water-filled plastic bottle (up to 15 cm long) was used for the adaptive version of adult bCPAP. The entire package (Annex 1) is produced adequate back pressure through expiratory bubbles to maintain the required positive end-expiratory pressure (PEEP). The bCPAP bottle and nasal seal was submerged in 70% isopropyl alcohol for 72 hours before the reuse.

Summary findings of design phase where adaptive bCPAP was first time used in five healthy individuals

Oxygenation using the adaptive version of the bCPAP was found to be comfortable and well tolerated by all five healthy adult participants. They reported mild form of discomfort at higher pressures (14 or 15cm PEEP). No significant differences of vitals were observed between before and after the intervention (Table 1). No adverse events including trauma/injury, erosion, bruise, bleeding, obstruction, breathlessness, pneumothorax, pneumomediastinum, abdominal distension during and after the trial were reported. There were no significant differences between anticipated and measured pressure (consecutive five measurements were done one minute apart) for each patient (Table 2), though there was 12% difference identified at the highest pressure (15 cmH_2O). The trial was thoroughly supervised by the principal investigator, the study physicians and engineers.

Thus, the design phase has demonstrated that (a) using a collaborative approach we can consistently produce adult silicone based nasal seals, (b) we can deliver effective PEEP with a mean pressure that is overall within +/-12% of target pressure, with most loss only at the highest pressure setting (15cmH_2O), (c) the device is well tolerated by participants with normal vitals and no adverse events.

Additional file 2. COREQ checklist.

COREQ (COnsolidated criteria for REporting Qualitative research) Checklist

A checklist of items that should be included in reports of qualitative research. You must report the page number in your manuscript where you consider each of the items listed in this checklist. If you have not included this information, either revise your manuscript accordingly before submitting or note N/A.

Topic	Item No.	Guide Questions/Description	Reported on Page No.
**Domain 1: Research team and reflexivity**			
Personal characteristics			
Interviewer/facilitator	1	Which author/s conducted the interview or focus group?	5
Credentials	2	What were the researcher’s credentials? E.g. PhD, MD	1
Occupation	3	What was their occupation at the time of the study?	1
Gender	4	Was the researcher male or female?	[illegible]
Experience and training	5	What experience or training did the researcher have?	5-6
Relationship with participants			
Relationship established	6	Was a relationship established prior to study commencement?	6
Participant knowledge of the interviewer	7	What did the participants know about the researcher? e.g. personal goals, reasons for doing the research	6
Interviewer characteristics	8	What characteristics were reported about the inter viewer/facilitator? e.g. Bias, assumptions, reasons and interests in the research topic	5-6
**Domain 2: Study design**			
Theoretical framework			
Methodological orientation and Theory	9	What methodological orientation was stated to underpin the study? e.g. grounded theory, discourse analysis, ethnography, phenomenology, content analysis	5
Participant selection			
Sampling	10	How were participants selected? e.g. purposive, convenience, consecutive, snowball	6
Method of approach	11	How were participants approached? e.g. face-to-face, telephone, mail, email	6
Sample size	12	How many participants were in the study?	6
Non-participation	13	How many people refused to participate or dropped out? Reasons?	6
Setting			
Setting of data collection	14	Where was the data collected? e.g. home, clinic, workplace	6
Presence of non-participants	15	Was anyone else present besides the participants and researchers?	6
Description of sample	16	What are the important characteristics of the sample? e.g. demographic data, date	5-6
Data collection			
Interview guide	17	Were questions, prompts, guides provided by the authors? Was it pilot tested?	6
Repeat interviews	18	Were repeat inter views carried out? If yes, how many?	N/A
Audio/visual recording	19	Did the research use audio or visual recording to collect the data?	6
Field notes	20	Were field notes made during and/or after the inter view or focus group?	5-6
Duration	21	What was the duration of the inter views or focus group?	6
Data saturation	22	Was data saturation discussed?	6
Transcripts returned	23	Were transcripts returned to participants for comment and/or	N/A

Topic	Item No.	Guide Questions/Description	Reported on Page No.
		correction?	
**Domain 3: analysis and findings**			
Data analysis			
Number of data coders	24	How many data coders coded the data?	7
Description of the coding tree	25	Did authors provide a description of the coding tree?	7
Derivation of themes	26	Were themes identified in advance or derived from the data?	7
Software	27	What software, if applicable, was used to manage the data?	N/A
Participant checking	28	Did participants provide feedback on the findings?	N/A
Reporting			
Quotations presented	29	Were participant quotations presented to illustrate the themes/findings? Was each quotation identified? e.g. participant number	N/A
Data and findings consistent	30	Was there consistency between the data presented and the findings?	10-11
Clarity of major themes	31	Were major themes clearly presented in the findings?	10-11
Clarity of minor themes	32	Is there a description of diverse cases or discussion of minor themes?	10-11

Developed from: Tong A, Sainsbury P, Craig J. Consolidated criteria for reporting qualitative research (COREQ): a 32-item checklist for interviews and focus groups. *International Journal for Quality in Health Care*. 2007. Volume 19, Number 6: pp. 349 – 357

Once you have completed this checklist, please save a copy and upload it as part of your submission. DO NOT include this checklist as part of the main manuscript document. It must be uploaded as a separate file.
